# Cerebral Abscess Due to Nocardia beijingensis Associated With HIV: Case Report and Mini Review

**DOI:** 10.7759/cureus.47571

**Published:** 2023-10-24

**Authors:** Cesar A Nieves Perez, María José Sánchez Pérez, Ana S Vargas, Maria A Franco, Miguel C Molina Obana

**Affiliations:** 1 Internal Medicine, Hospital Angeles Pedregal, Mexico City, MEX; 2 Medicina Interna, Hospital Angeles Pedregal, Mexico City, MEX; 3 General Medicine, Universidad del Valle de Mexico, Hermosillo, MEX

**Keywords:** general internal medicine, infectology, infec, hiv-associated infection, nocardia brain infection, brain abscess

## Abstract

Brain abscesses are severe focal infections of the central nervous system. We report the case of a 37-year-old patient with a recent diagnosis of HIV, who presented with weakness in the left arm that progressed to left hemiplegia, ipsilateral paresthesia, holo cranial headache, fever accompanied by chills, and left tonic-clonic movements. A craniectomy and lesion resection were performed along with antimicrobial treatment. Subsequently, the patient persisted with left hemiplegia, which significantly improved after the procedure and gradually through physical physiotherapy. During the investigation, we complete medical history, physical examination, Image tests, laboratory tests, and cultures. After the finalization of the approach, the final diagnosis was a brain abscess due to Nocardia beijingensis associated with HIV. The patient was managed with anticonvulsants: levetiracetam, antimicrobials: ceftriaxone, trimethoprim/sulfamethoxazole, metronidazole, and vancomycin, Craniotomy plus resection of two brain abscesses, Steroidal anti-inflammatory: dexamethasone and antiretroviral therapy. With this, the patient was discharged successfully from the hospital.

## Introduction

Brain abscesses are infectious lesions focused on the central nervous system, characterized by areas of cerebritis and central necrosis surrounded by a highly vascularized capsule [1]. Brain abscesses usually account for approximately 8% in developing nations and 1-2% in developed countries among intracranial space-occupying lesions. They more commonly appear in the male age group of 30-50 years and are associated with a mortality rate of 50-55% [1,2]. In a significant percentage of cases, the primary etiology is polymicrobial, although the most commonly isolated agents are Streptococcus viridans and Staphylococcus aureus [2]. However, in HIV patients, the prevalence and incidence of central nervous system infections, specifically in those with a CD4 count between 200-500 cells/microliter and without retroviral treatment, opportunistic infections such as toxoplasmosis encephalitis or brain abscess secondary to Staphylococcus spp, Streptococcus spp, Salmonella spp, Aspergillus spp, Nocardia spp, and tuberculomas, among others, should be considered. Nocardia is a gram-positive, branching, aerobic bacterium, ubiquitously present and acid-alcohol resistant, found in water and decomposing plants [3]. In the case of abscesses caused by Nocardia, there is currently limited information, and most of the existing data consists of reports and case series. It is estimated that approximately 2% of brain abscesses are caused by Nocardia, which tends to occur not in immunocompetent patients but rather in immunocompromised patients with risk factors such as immunosuppressive treatment, premedication, hematological neoplasms, organ transplantation, HIV infection, chronic lung diseases, diabetes, and chronic kidney disease [3,4]. It is often associated with a high morbidity and mortality rate of around 33%, which is three times higher than that caused by other etiologies, given that the overall mortality rate of abscesses is 10% [5]. This type of abscess is typically managed through combined medical therapy and surgery with craniotomy and excision, as this approach has been shown to be associated with a better prognosis in the majority of cases [5].

## Case presentation

A 37-year-old male patient with no significant chronic degenerative history presented with symptoms that began one month before hospital admission. He initially experienced progressive weakness in the left upper limb, accompanied by paresthesias that progressed to hemiplegia of the left upper limb and ipsilateral facial paresthesia. He also developed gait lateralization and decreased strength in the left lower limb. One week before hospital admission, he started experiencing a holo cranial oppressive headache (6/10 on the pain scale) associated with fever and multiple episodes of vomiting. He sought medical attention, and a brain MRI revealed an intra-axial lesion at the superior right parietal cortico-subcortical level. These lesions were associated with disproportionate vasogenic edema and moderate mass effect. The primary diagnostic consideration was multiple cerebral abscesses (early stage) vs neoplastic activity as a secondary possibility. He was started on prednisone 40 mg every 24 hours to reduce the edema.

**Figure 1:**
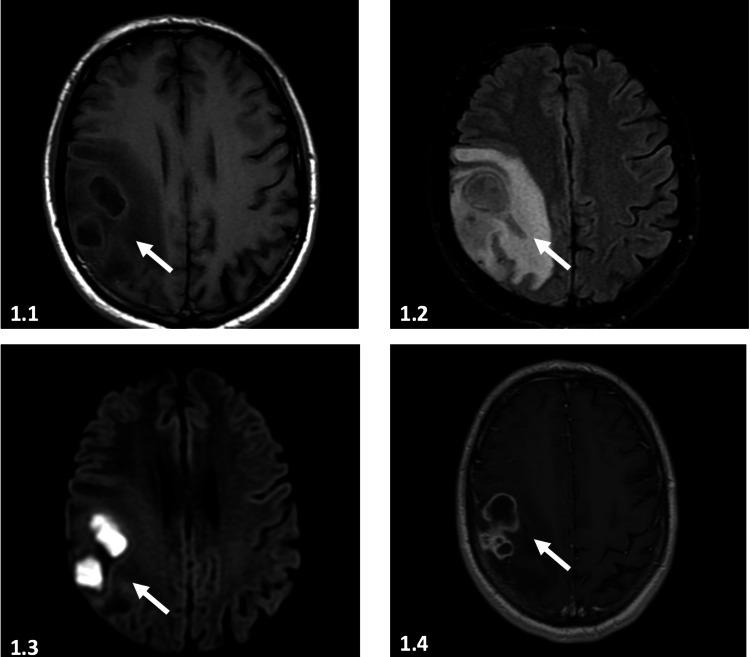
Brain MRI A) T1 sequence: space-occupying process, hypointense with isointense wall and significant vasogenic edema located in the right precentral gyrus. B) T2 FLAIR sequence: lesion with double wall image that is hyperintense in its internal wall and isointense in its external wall with vasogenic edema, obliteration of the subarachnoid space. C) Diffusion sequence: significant and homogeneous increase in the signal intensity of the central area of ​​the lesion. D) Contrast-enhancedT1 sequence: annular, intense reinforcement with an irregular but well-defined contour of its wall.

Further evaluation was performed with contrast-enhanced MRI, which showed nodular, confluent intra-axial lesions in the white matter of the right frontal second convolution (Figure 1). These lesions demonstrated restricted diffusion in their central portion and presented a lactate peak in spectroscopy. Cerebral abscess was the leading diagnostic impression. On the day of hospital admission, the patient's neurological condition deteriorated, characterized by tonic-clonic movements in the left arm. Treatment was initiated with levetiracetam 1.5 g orally and he was referred to the emergency department.

In the emergency department treatment with levetiracetam was continued and new treatment with dexamethasone, paracetamol, ceftriaxone, metronidazole (for seven days), trimethoprim-sulfamethoxazole, and vancomycin (for eight days) was started. Hemocultures were taken, and a simple CT scan without contrast of the brain was performed for neuronavigation purposes before the procedure. A craniotomy was performed for resection of two cerebral abscesses, with tissue cultures revealing Nocardia beijingensis. Following the procedure, the patient showed progressive improvement in motor symptoms, regaining ambulation, and motor function in both the thoracic and pelvic limbs. Due to the absence of fever and this improvement, the patient was transferred to a standard ward for ongoing management.

The patient had a positive rapid HIV test conducted one week before admission, prompting further confirmatory diagnostic evaluation. The viral load was measured at 3 million copies (normal range: undetectable), and the CD4 count was 34 cells/mm^3 (normal range: CD4 500 to 1500 cells/mm^3). After discharge, the patient initiated antiretroviral therapy with a combination of a nucleotide analog reverse transcriptase inhibitor (tenofovir and efavirenz) and an integrase inhibitor (dolutegravir).

## Discussion

Microbiology and epidemiology

Nocardia was initially isolated and named by Edmond Nocard in 1888 in a case of bovine lymphadenitis. Subsequently, it was isolated by multiple scientists throughout history, who encountered various difficulties in classifying it correctly. Currently, its taxonomy is described based on its molecular and antimicrobial resistance characteristics. The Nocardia group is associated with the actinomyces group and specifically belongs to the family Mycobacteriaceae. They are gram-positive, branching, weakly acid-alcohol resistant bacilli that fragment into cocobacillary forms. There are currently 22-30 described species, of which 16 are associated with human diseases [6,7]. Among the taxonomic group of Nocardia, the most significant pathogens are members of the former N. asteroides complex. Nocardia is widely distributed throughout the world with varying prevalence, as a saprophyte and innocuous organism found in soil, water, and decaying plant material. However, its isolation has been found in common sources like household dust, swimming pools, and tap water. Infection usually occurs through two routes: direct inoculation into skin and soft tissues, or ingestion and inhalation, with the latter being the most common mode of transmission. Although it is a well-known cause of infection in animals, especially in cattle, there has been no documented transmission from animals to humans. In cases of transmission between humans, this has been described anecdotally in oncology and transplant units [5,7]. Nocardiosis infections are often observed in patients with conditions that compromise the immune system, such as diabetes, HIV, autoimmune diseases, innate immunity disorders, post-transplant patients, malignancies, and those undergoing prolonged corticosteroid treatments [6,8].In some cases, infections have been reported in patients with environmental exposure, such as those involved in gardening, agriculture, and even healthcare workers exposed to surgical site infections and catheter-associated infections. In immunocompromised patients, the pulmonary manifestation is more prevalent, with hematogenous dissemination to the lungs and cerebral cortex. In immunocompetent patients, cutaneous infection is more common [6,8]. Currently, there is limited information on the disease epidemiology, but it has a global reach, with higher prevalence in tropical regions such as India, Pakistan, Iran, Canada, Spain, Australia, Latin America, and the southern United States. Its incidence appears to increase each year due to population growth, a higher number of immunocompromised patients, and increased awareness. In the United States, approximately 500-1000 cases are reported annually, and in Italy, 90-130 cases are reported. In HIV patients, nocardiosis is reported in 3.4-16.7% of patients, especially in those with a CD4 count of 50 cells.

Clinical presentation

Associated with Nocardia Infection: The hallmark of nocardiosis is the formation of abscesses, with a chronic course tending to recur or cause relapses despite adequate medical therapy. It has been isolated in different anatomical sites depending on the geographic region, but the most common site of infection is the lung, occurring in two-thirds of patients. Extrapulmonary involvement can affect bones, skin, eyes, heart, joints, and kidneys, manifest as catheter-related bacteremia, disseminated nocardiosis, or healthcare-associated infections. Among the extrapulmonary manifestations, the central nervous system is the most common, with a prevalence of 20-58%, being more frequent in immunocompromised individuals. In the central nervous system, nocardiosis often presents with acute or subacute neurological deficits, headaches, seizures, or ataxia. Local effects may result from the appearance of granulomas or abscesses in the brain, spinal cord, or meninges. This invasion can be cryptic, persisting with varying signs and symptoms ranging from slow to rapid progression, with a wide variety of neurological deficits that can be mistaken for tumors [8]. A study conducted by Corsini Campioli et al. analyzed a population of 24 patients with brain abscesses to describe their clinical presentation and outcomes. The most common comorbidities in patients were chronic kidney disease (45%), hypertension (33.3%), and diabetes mellitus (29.1%). Pulmonary and cutaneous infections were the most common primary sites of infection (37.5% and 12.5%), with the most common location of abscess formation being the frontal lobe (41.6%) [9].

Diagnosis

The diagnosis of abscesses secondary to Nocardia spp. is primarily performed through culture isolation. Commonly used stains include Gram stain and modified acid-fast stains (e.g., Kinyoun stain). Gram-positive filaments are observed, which are thin, beaded, and branched at right angles. With the modified acid-fast Kinyoun stain, Nocardia is often seen as partially acid-fast filamentous bacilli [6]. Molecular tests, such as PCR or genomic sequencing, are not routinely performed due to their limited availability. However, when the pathogen cannot be isolated using traditional microbiological methods and clinical suspicion persists, referral to a specialized center for PCR or genomic sequencing is recommended. Furthermore, there are no pathognomonic histological findings for Nocardia, but microscopic visualization of tissue necrosis with infiltrates of polymorphonuclear cells, lymphocytes, plasma cells, and macrophages is highly suggestive.

Treatment

There is varying antimicrobial susceptibility among different species of Nocardia, which depends on prevalence and the country where the study is conducted. In a retrospective study of 765 isolates by the CDC between 1995 and 2004, 42% were found to be resistant to trimethoprim-sulfamethoxazole (TMP-SMX), and 61% were resistant to sulfamethoxazole [10]. In contrast, a study of 552 bacterial isolates collected from 6 reference centers in the United States between 2005-2011 showed only 2% resistance to TMP-SMX and/or sulfamethoxazole [11]. According to the CDC, the discrepancy between these results lies in the study design, susceptibility testing methods, and laboratory interpretation. However, other studies conducted in France [12] and Taiwan [13] showed consistency with the results of the 552 isolates, with 2% resistance to TMP-SMX. Therefore, susceptibility to TMP-SMX is reported in the vast majority of Nocardia species. N. farcinica is uniformly susceptible to amikacin and TMP-SMX but generally resistant to third-generation cephalosporins. N. nova, on the other hand, is susceptible to TMP-SMX, third-generation cephalosporins, clarithromycin, imipenem, and amikacin. N. brasiliensis is typically susceptible to TMP-SMX and amikacin, with 87% susceptibility to third-generation cephalosporins and only 20-40% susceptibility to imipenem. N. cyriacigeorgica shows resistance to amoxicillin/clavulanic acid, unlike N. abscessus. N. otitidiscaviarum is resistant to imipenem, and N. transvaalensis is usually resistant to amikacin and other aminoglycosides [14, 15]. Susceptibility to carbapenems depends on geographical region, Nocardia species, and intrinsic differences among carbapenems. Susceptibility to imipenem is reported to be from 30 to 90%, compared to ertapenem, which reports susceptibility 16 times lower than imipenem [16]. However, an effective specific therapy for nocardiosis has not been determined. Therefore, empirical treatment with TMP-SMX, considered standard therapy, is recommended [17, 18].

## Conclusions

Brain abscesses caused by Nocardia are an unusual yet serious manifestation of infection, primarily affecting immunocompromised individuals. While they represent a relatively small proportion of overall brain abscess cases, their high morbidity and mortality make their diagnosis and treatment crucial. They are characterized by the formation of abscesses with a chronic and recurrent tendency, which complicates management. The infection can affect various extrapulmonary sites, but the central nervous system is the most commonly affected, presenting with acute or subacute neurological symptoms. Diagnosis relies on culturing and isolating Nocardia spp., and although molecular tests could be helpful, their availability is limited. The treatment of cerebral nocardiosis presents challenges due to the variability in antimicrobial susceptibility among different Nocardia species. Empirical therapy with trimethoprim-sulfamethoxazole (TMP-SMX) is considered standard, but resistance to this antimicrobial and others has also been documented. Selecting the appropriate treatment should be based on susceptibility test results and should consider the specific Nocardia species involved, which in selected cases should be accompanied by surgical intervention. Therefore, cerebral nocardiosis is a significant clinical entity that demands a multidisciplinary approach, involving multiple specialists to achieve proper management.
